# Efficiency of ultrasonic retrieval for separated instruments within the middle third of root canals using modified staging platform: a comparative in-vitro study

**DOI:** 10.1186/s12903-026-08029-8

**Published:** 2026-03-25

**Authors:** Basim Samir Mohamed, Nihal Ezzat Sabet, Dina Ahmed Morsy

**Affiliations:** Faculty of Dentistry, Department of Endodontics, Cairo University, Cairo, Egypt

**Keywords:** Conventional staging platform, Loop retrieval, Modified staging platform, Separated instruments, Ultrasonic retrieval

## Abstract

**Aim:**

This study aimed to compare the efficiency of ultrasonic retrieval using a Modified Staging Platform (MSP) versus a Conventional Staging Platform (CSP) for 3, 5, and 7 mm separated NiTi instrument fragments in moderately curved mesiobuccal canals of mandibular molars, by assessing retrieval time, success rate, and vertical root fracture resistance, with loop intervention for unretrieved fragments.

**Methodology:**

Seventy-seven extracted mandibular first molars were prepared and allocated randomly into 2 main groups (CSP, MSP; *n* = 33 each) and one control group (*n* = 11). Each main group was subdivided into 3 subgroups (*n* = 11) according to separated fragment-length (3, 5, 7 mm). NiTi rotary instruments were separated in mesiobuccal canals, and retrieval was attempted under dental operating microscope (DOM) using CSP or MSP corresponding to the allocated group. Retrieval time and success rate (maximum 45 min) were recorded for each case. Failed samples underwent loop retrieval (15 min). Following retrieval procedures, mesial roots were sectioned and decoronated to a standardized root length (15 mm), for vertical fracture testing using a universal testing machine. Nonparametric tests (Mann–Whitney U and Kruskal–Wallis with Dunn post hoc) were applied (α = 0.05).

**Results:**

For 3 mm fragments, MSP yielded significantly shorter ultrasonic retrieval time than CSP (*p* < 0.001). For 5 mm fragments, CSP was faster than MSP (*p* = 0.002). Neither technique retrieved 7 mm fragments ultrasonically. Loop retrieval succeeded in 90.9% of 7 mm fragments for CSP but failed for MSP (*p* < 0.001). Both retrieval methods showed significantly lower fracture resistance than controls (*p* < 0.001); no significant difference in fracture resistance was found between MSP and CSP groups.

**Conclusion:**

MSP was more time-efficient than CSP for short (3 mm) fragments, whereas CSP performed better for 5 mm fragments and permitted successful loop-assisted removal of long (7 mm) fragments. Both techniques significantly reduced vertical root fracture resistance relative to intact controls.

**Graphical abstract:**

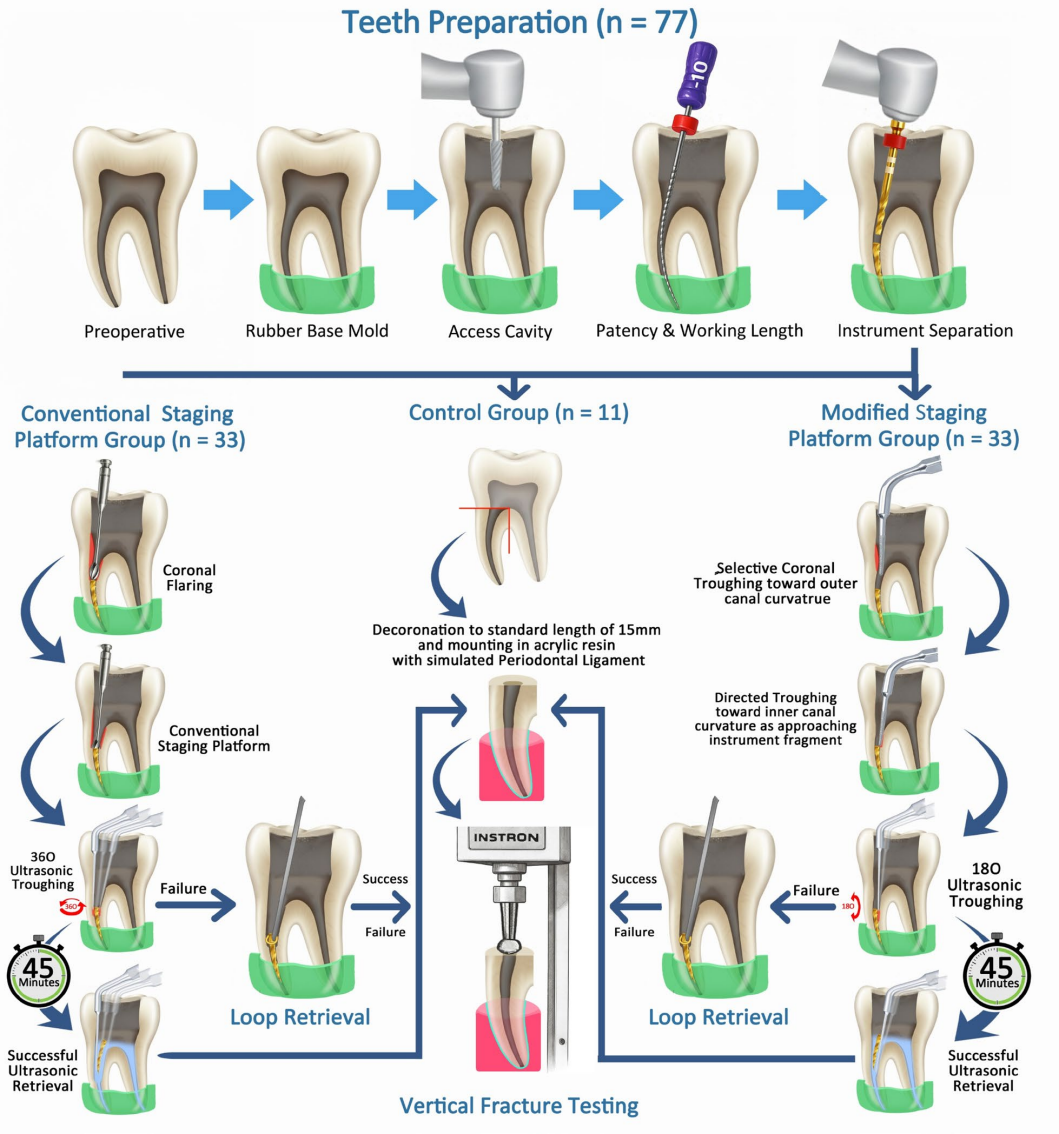

## Introduction

Nickel-titanium (NiTi) rotary instruments have significantly enhanced endodontic practice owing to their flexibility and ability to produce a centred root canal preparation, thereby lowering the risk of procedural errors [1]. Despite these advantages, instrument separation remains a recognized complication that may occur without prior warning, with incidence ranging from 0.39% to 5% [2]. As separated fragment may obstruct canal disinfection and compromise treatment outcomes, its management represents a significant clinical challenge.

Management strategies generally include retrieval or bypassing the separated fragment, based on factors such as fragment location, canal curvature, and patient-specific anatomy [3]. Retrieval is often preferred when feasible, as regaining full canal accessibility enhances decontamination of the root canal space and long-term treatment success [2, 4]. In contemporary practice, integration of ultrasonic (US) techniques with the dental operating microscope (DOM) has become the standard protocol, substantially enhancing retrieval outcomes [5]. US retrieval assisted by a DOM yields success rates within the range of 33% to 100%, largely depending on fragment location, retrieval strategies, and visibility of the separated instrument [6].

A critical step in ultrasonic retrieval procedures is the creation of straight-line access to fragment. Conventionally this is achieved using modified Gates Glidden (GG) to create a Conventional Staging Platform (CSP). The CSP widens the canal coronally into a funnel shape, which enhances the operator’s ability to visualize the fragment under an operating microscope with a rim of dentin of 360° all around. CSP creates sufficient space for the introduction of US tips, which are subsequently applied to trephine the dentine encircling the fragments, facilitating their disengagement from the canal [4, 7]. Previous studies have shown that CSP-assisted ultrasonic retrieval under magnification significantly improves the likelihood of fragment removal, particularly when the fragment is located in a straight canal segment [8, 9]. However, the success of CSP decreases when the fragment is situated in the curved region. The requirement of the 360° dentin removal, especially with the use of large and rigid GG drills, may result in excessive dentin sacrifice in areas of reduced dentin thickness. This increases the risk of procedural complications such as canal transportation, strip perforation, and structural weakening, potentially reducing vertical fracture resistance [10, 11]. Consequently, while CSP may enhance retrieval success, its biomechanical implications raise concerns in anatomically complex canals.

To address these limitations, Narasimhan et al., 2021 [12], proposed a Modified Staging Platform (MSP) created utilizing ultrasonically driven tips to expose only 180° of dentin around the separated fragment, directed toward the inner wall of the curvature. This approach helps minimize canal damage and improve reestablishment of canal patency, particularly when instrument fragments are located in the middle third of curved canals. Therefore, the MSP is not merely an alternative access method, but a biomechanically informed strategy designed to mitigate the well-documented iatrogenic consequences of traditional techniques, thereby improving the potential for re-establishing canal patency while conserving tooth structure [12, 13].

Beyond access design and canal curvature, fragment length significantly influences retrieval outcome. The US retrieval becomes less predictable when fragment length exceeds approximately 4.6 mm, as their removal frequently demands the sacrifice of additional dentin to expose a sufficient coronal segment [14]. In such cases, the loop technique may present a mechanical alternative by engaging the exposed coronal end of the fragment and facilitating its withdrawal with minimal further dentin sacrifice and procedural risks [15]. Clinically, this technique may serve as a valuable adjunct when US retrieval alone is unsuccessful or when structural integrity is a priority.

Several studies have evaluated the effect of US retrieval of separated instruments located in the middle third of root canals using the CSP, reporting that this approach leads to an increase in canal volume and a corresponding reduction in vertical root fracture resistance [16, 17]. However, no studies have compared the MSP to CSP at various retrieval lengths in the middle third. Additionally, the impact of MSP on vertical fracture resistance has not been yet evaluated, and the effectiveness of the loop technique for retrieving fragments following failed US retrieval has not been systematically evaluated.

Therefore, the present study sought to assess the time and success rate of US retrieval of separated instruments measuring 3, 5, and 7 millimeters (mm) in length, as well as the resulting vertical root fracture resistance. This evaluation was conducted by comparing MSP versus CSP techniques in moderately curved mesial roots of mandibular molars. Additionally, the loop technique was employed to retrieve fragments when US retrieval was unsuccessful.

The null hypothesis was that no significant differences would be observed between MSP and CSP techniques in terms of US retrieval time, success rate, or vertical root fracture resistance for 3-, 5-, and 7-mm separated instruments in moderately curved mesial roots of mandibular molars, even when the loop technique was used following failed US retrievals.

## Materials & Methods

**Fig. 1:**
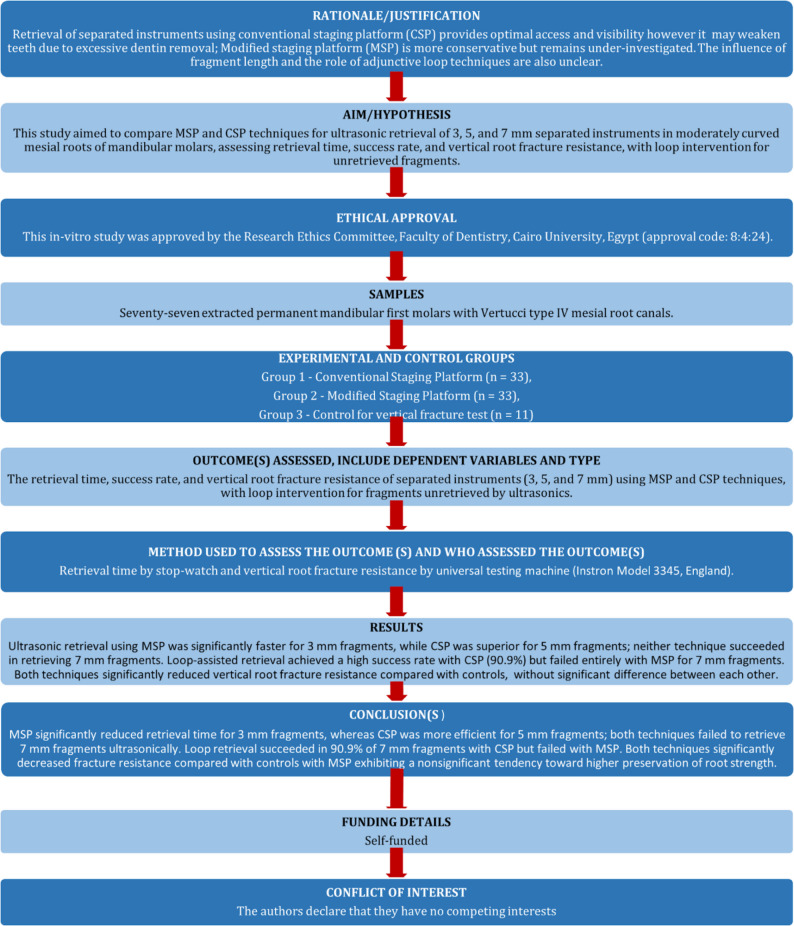
PRILE 2021 flowchart illustrating the research process

The manuscript of this laboratory study has been written according to Preferred Reporting Items for Laboratory studies in Endodontology (PRILE) 2021 guidelines [18] (Fig. 1).

This in-vitro study was conducted in the Endodontic Department, Faculty of Dentistry. Approval was obtained from Research Ethics Committee, Cairo University, Faculty of Dentistry (Approval code: 8:4:24). All teeth were collected from an anonymized pool of freshly extracted teeth from the Department of Oral and Maxillofacial Surgery at Faculty of Dentistry, Cairo University. Individuals who contributed teeth, or their legal guardians when appropriate, provided voluntary participation and previously consented to use their teeth for research purposes. This study was conducted in accordance with the principles of Helsinki’s declaration, as applicable to research involving human-derived materials.

**Fig. 2:**
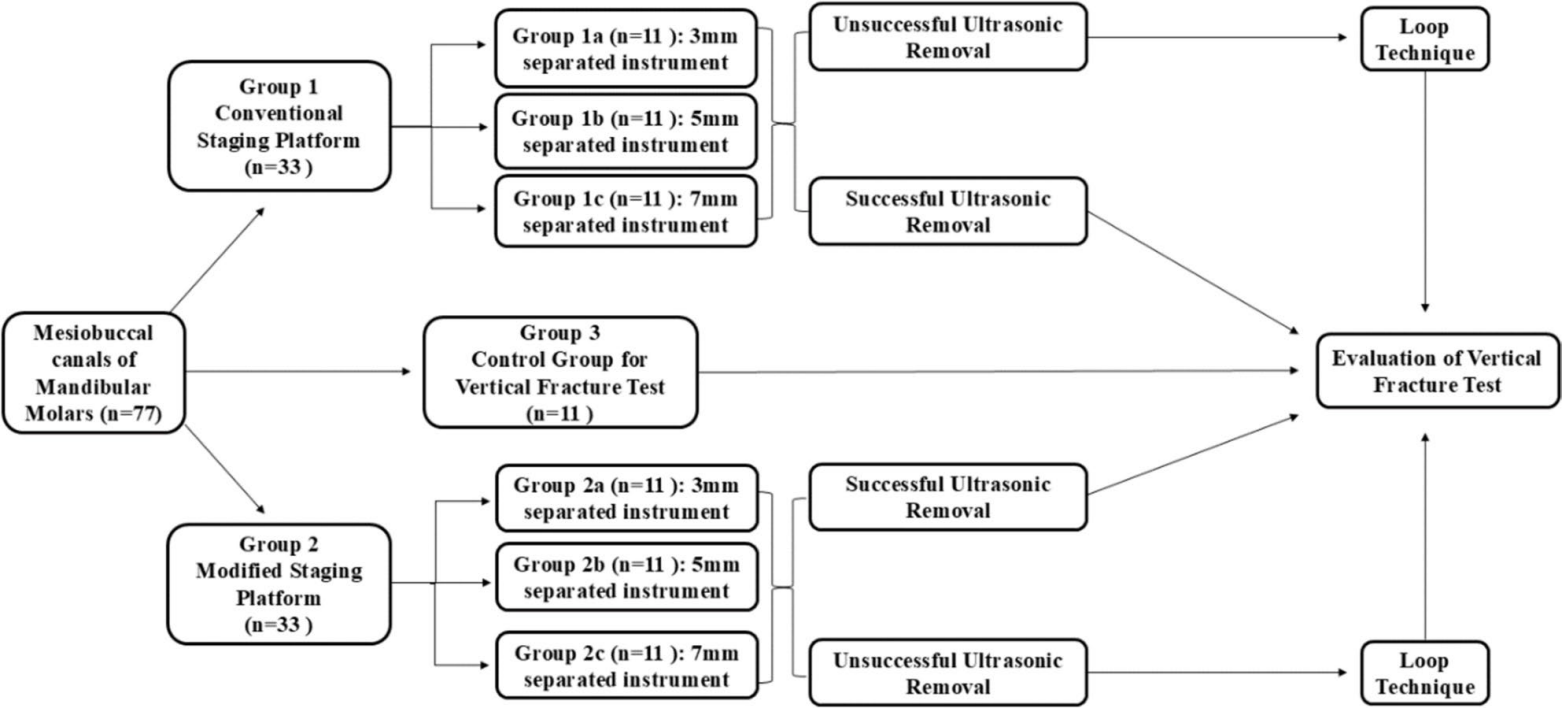
Diagrammatic representation of experimental workflow

A diagrammatic representation of experimental workflow is shown in Fig. 2.

## Study settings

### Selection and preparation of samples

Seventy-seven human permanent mandibular first molars, freshly extracted for periodontal or prosthodontic reasons, were obtained. Teeth were selected based on the following criteria: mature root apices, root length of 20–24 mm, mesial roots with a root canal configuration of type IV according to the Vertucci classification [19], moderately curved mesiobuccal roots 10°- 20° [20], no previous endodontic treatment, absence of anatomical anomalies, root resorption, fractures, cracks, or canal calcifications. Canal volume was not measured, as anatomical variability was minimized through these strict eligibility criteria, consistent with previous in vitro studies [13, 17]. The PS program was used to determine the sample size in accordance with findings of Shahabinejad et al. 2013 [9]. A sample size of 33 teeth per group was suitable for the study in terms of the primary outcome, retrieval time in minutes, with 80% power and a 0.05 error probability. A total of 77 teeth were included in the sample since each group was further divided into three subgroups (*n* = 11), with an extra 11 teeth added to act as the control group in the fracture resistance test. The external root surfaces of the selected teeth were carefully debrided with a curette to eliminate calculus deposits and residual periodontal ligament tissue, disinfected in 2.5% sodium hypochlorite (NaOCl) (Cerkamed Medical Company, Stalowa Wola, Poland) for 15 min, and cleaned ultrasonically (Suprasson P5 Booster, Satelec) to eliminate residual soft tissues. Samples were stored in 0.9% saline solution (Baxter International Inc., Deerfield, IL, USA) until experimentation. Initial screening was performed under the DOM (SEILER MEDICAL, St. Louis, Missouri) and confirmed radiographically from both mesio-distal and bucco-lingual aspects (digital periapical films (Soredex, Helsinki, Finland). Curvature measurements were verified using SCANORA software (Soredex, Helsinki, Finland).

All procedures were conducted by a single endodontist. Under DOM at 6× magnification, access cavity preparation was performed using high-speed diamond round burs and Endo-Z burs (Dentsply Maillefer, Ballaigues, Switzerland). Biomechanical Instrumentation of the mesiobuccal canals was initiated with a size #10 ISO K-file (MANI, INC., Industrial Park, Japan) to verify patency and establish the working length. The working length was set 1 mm short of the apical foramen following initial file inspection at the apex. Glide path preparation was subsequently carried out with #10 and #15 K-files. Coronal flaring was achieved using an MG3 Gold orifice opener file (#19/0.10) (Perfect^®^ Medical Instruments Co., Ltd, Shenzhen, China) with the X-smart motor (Dentsply Maillefer, Ballaigues, Switzerland). Canal preparation to the full working length was completed using only the MG3 Gold #20/0.04 rotary file. The subsequent #25/0.06 file was then manually verified to engage securely within the middle third, without reaching the full working length. Any sample not meeting this criterion was discarded. Following each enlarging instrument, the canals were irrigated with 5 mL of 2.5% sodium hypochlorite using a 30-gauge side-vented irrigation needle (Ultradent Products Inc., South Jordan, UT, USA), adjusted 2 mm shorter than the working length. From the 77 samples, 11 were randomly assigned to the control group, considered complete at this stage, and stored until their use in the fracture resistance test.

A total of 66 mandibular first molar teeth were randomly assigned into two main groups (*n* = 33 each) according to the type of staging platform prepared (conventional (CSP) or modified staging platform (MSP)). Then each of the two main groups was further subdivided into three subgroups (*n* = 11 each) according to length of instrument fragment to be separated whether 3, 5, or 7 mm.

### Intracanal separation of instruments

**Fig. 3:**
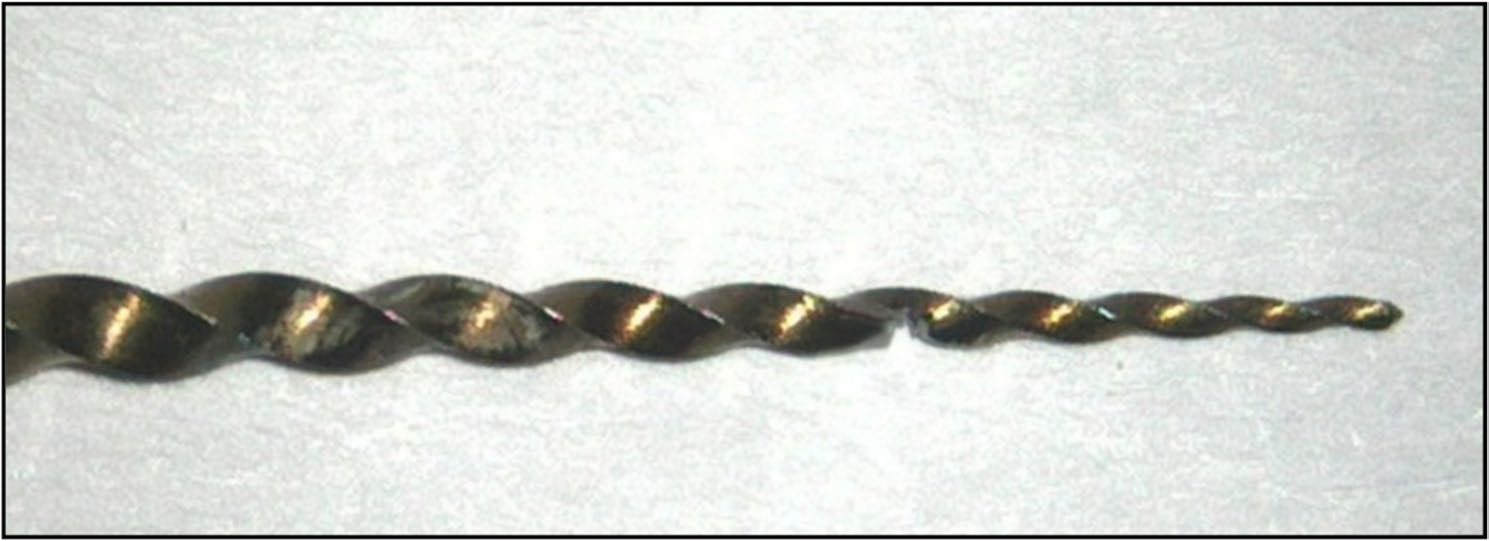
The rotary file notched to half thickness at 5 mm from the tip

For each molar, MG3 Gold rotary file (#25/0.06) was partially notched to half its thickness using a 0.3-mm diamond disc mounted on a low-speed handpiece to achieve controlled separation. The notch is positioned to be at 3, 5, or 7 mm from the tip according to fragment designated subgroup (Fig. 3). The rotary file was then mounted to X-smart motor and placed into the middle third of mesiobuccal canal and operated at 350 rpm with 3 N torque. The file was rotated with pressure until fracture occurred, after which a periapical radiograph confirmed fragment location in the middle third of the canal, with its coronal end at the predetermined depth. Any sample where the fragment was positioned outside this zone was excluded.

### Ultrasonic retrieval procedures

Each tooth specimen was secured in a rubber base mold (Zhermack SpA, Badia Polesine, Italy), maintaining controlled vertical and horizontal alignment to allow efficient handling during retrieval. Retrieval procedures were conducted under magnification of 11x of DOM.

### Conventional Staging Platform (CSP)

#### Establishing radicular access and CSP

The retrieval procedure was conducted according to the technique described by Ruddle, 2004, aiming directly at the top of the instrument fragment. The coronal flaring to the separated instrument was achieved using GG drills (Mani Inc., Tochigi, Japan) sizes #2 and #3. Next, a conventional staging platform was created using a modified GG size #3 (Mani Inc., Tochigi, Japan). The modification was done by grinding the drill’s cutting end at its maximum cross-section. Platform was achieved when the modified GG size #3 (Mani Inc., Tochigi, Japan) contacted the most coronal part of the separated instrument, exposing a rim of dentin around its entire circumference [21].

#### Instrument retrieval

**Fig. 4:**
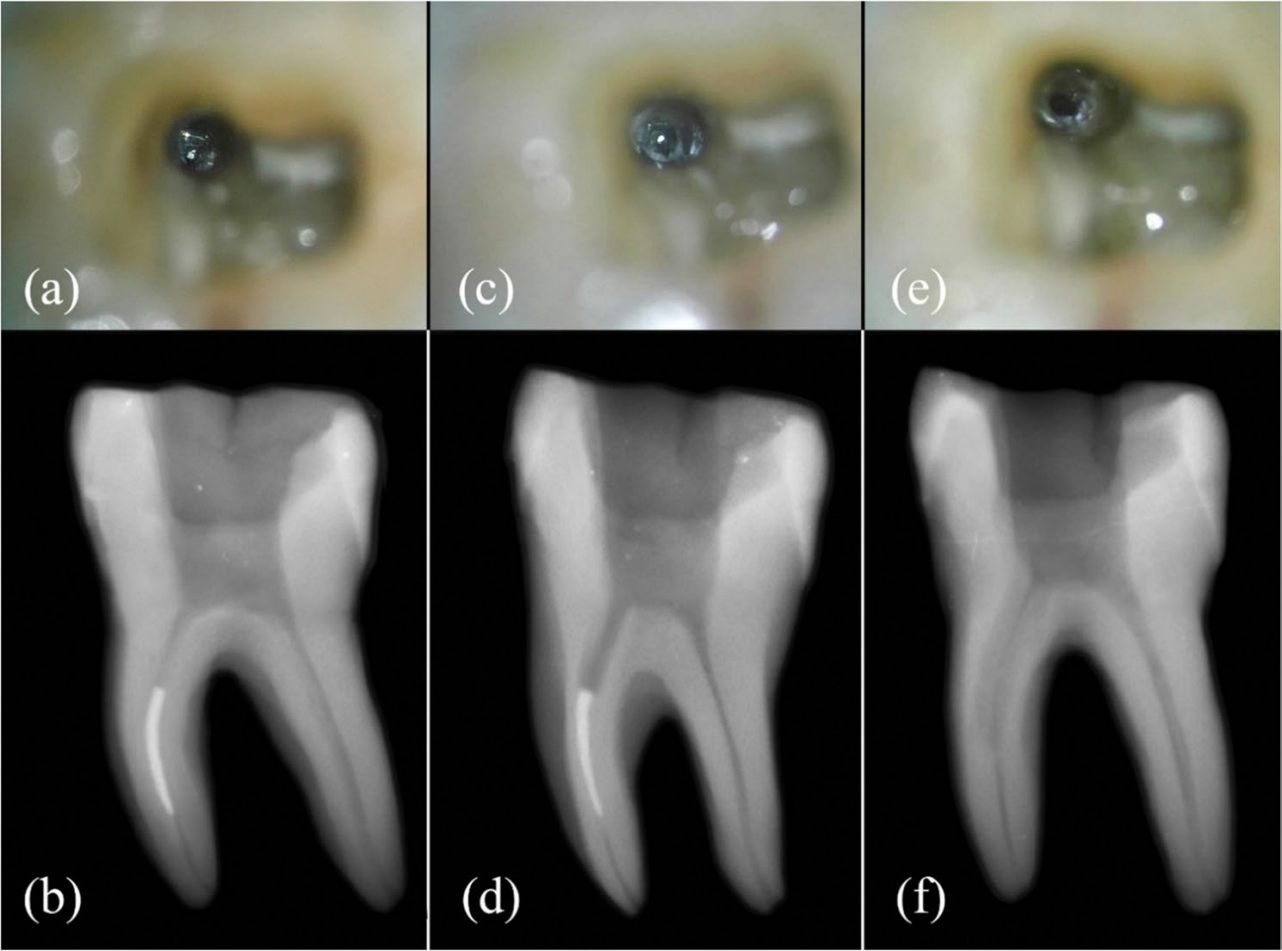
Conventional staging platform (**a**) Clinical photograph of separated instrument fragment separated in middle third of mesiobuccal canal under 11x magnification of DOM, (**b**) pre-operative radiograph, (**c**) conventional staging platform was created at 360° around instrument fragment, (**d**) intraoperative radiograph of the platform, (**e**) mesiobuccal canal after retrieval of the instrument fragment, (**f**) post-operative radiograph to confirm complete removal of the instrument fragment without any retained metal fragment

A smooth tapered US tip ET25 (Satelec, Merignac, France) driven by a piezoelectric unit (Acteon, Satelec, France) used at low power setting (40% of maximum device at E-mode) in dry condition to trephine the dentin along the full circumference of the separated instrument in a counterclockwise motion, exposing its coronal 2–3 mm until the file became loosened. Loosening was confirmed when the instrument could be relocated from its position using a DG-16 (Hu-Friedy Mfg Co, LLC, Chicago, IL). Subsequently, 17% EDTA liquid was injected into the canal. The power of the US device was then increased by 30%, reaching a total power of 70%. The ET25 US tip was placed between the fragment and canal wall and activated with counterclockwise strokes in the presence of 17% EDTA. The resulting ultrasonic vibrations and acoustic streaming facilitated loosening and subsequent disengagement of the instrument from the canal (Fig. 4).

### Modified Staging Platform (MSP)

#### Establishing radicular access

The MSP procedure was conducted according to the technique described by Narasimhan et al., 2021 [12], approaching the separated instrument at a slight angle. To establish a radicular access, the angle is formed by positioning the coronal aspect of the radicular access pathway against the outer wall of the root canal, and the apical aspect is placed above the instrument fragment against the inner wall of the curvature. The radicular access was initiated from the orifice level with preferential cutting toward the outer wall of curvature using a thin, tapered, diamond-coated US tip ET20D (Satelec, Merignac, France) without coolant. ET20D was progressed toward the head of the fragment, with the US tip activated in intermittent vertical motions. As the ET20D moved apically toward the tip of the separated fragments, it was carefully directed to stop precisely at the head of the separated fragment, resting against the inner curvature wall. Radicular access was considered complete when the top of the fragment became visible along its entire circumference [12].

### Establishing the MSP

The MSP, approximately 180°, was created around the instrument fragment, directed toward the inner curvature wall, using the same US tip ET20D in dry conditions. This process exposed a rim of dentin, which facilitated the placement of the subsequent US tip.

#### Instrument retrieval

**Fig. 5:**
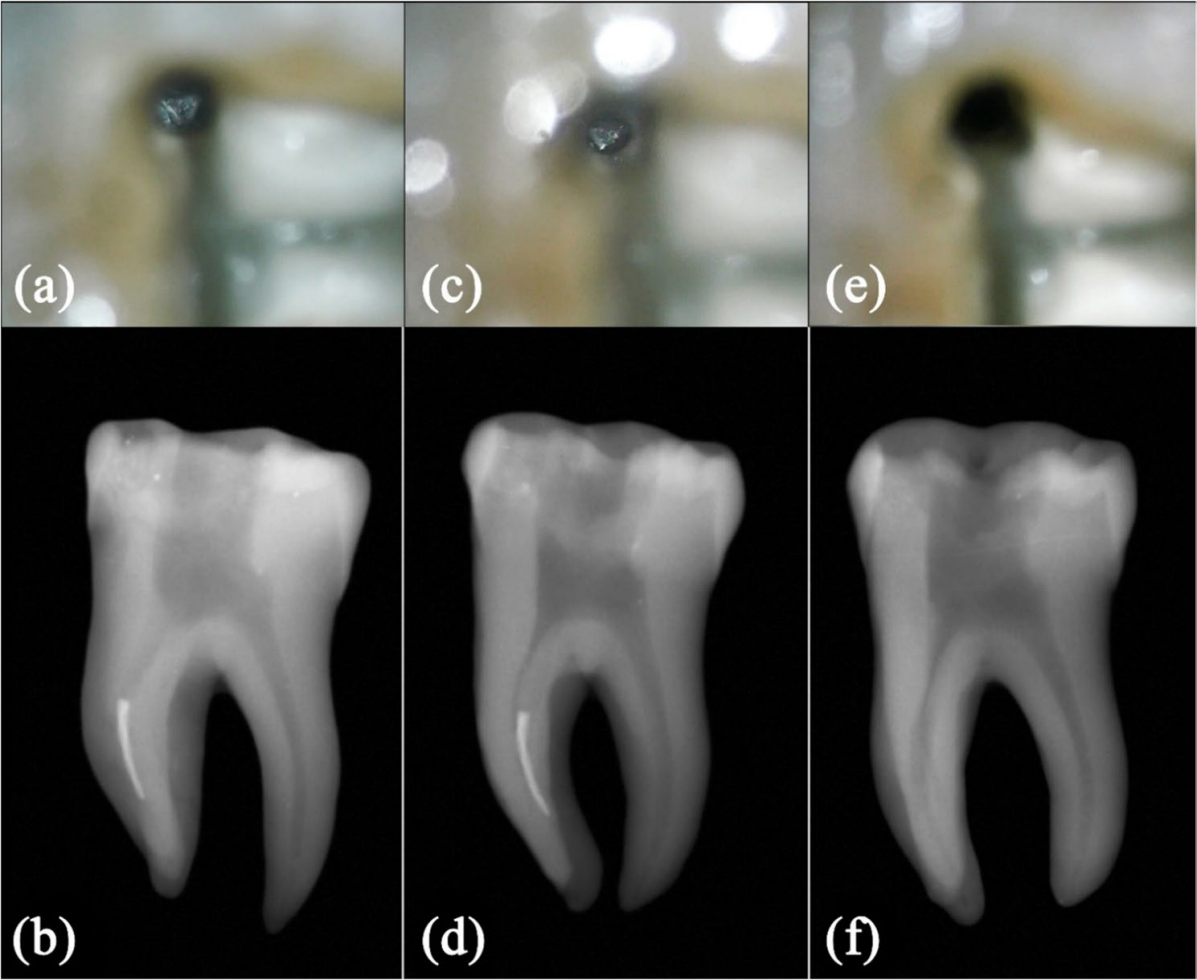
Modified staging platform (**a**) Clinical photograph of separated instrument within mesiobuccal canal under 11x magnification of DOM, (**b**) preoperative radiograph, (**c**)modified staging platform was created at 180° along inner canal wall, (**d**) intraoperative radiograph of the modified staging platform, (**e**) mesiobuccal canal after retrieval of instrument fragment. (**f**) postoperative radiograph to confirm complete removal of the instrument fragment without any retained metal fragments

A smooth, tapered US tip ET25 was then used at low power setting (40% of maximum device at E-mode) in dry conditions to trephine the dentin along only 180° of the inner walls of the separated instrument by vertical strokes to expose 2–3 mm of the instrument fragment until the instrument was loosened. Canal irrigation using 17% EDTA was performed. The power of the US device was then increased by 30%, reaching a total power of 70%. The ET25 US tip was positioned in the space separating the fragment from inner canal wall and activated with vertical strokes in the presence of 17% EDTA. The resulting ultrasonic vibrations and acoustic streaming facilitated loosening and subsequent disengagement of the instrument from the canal (Fig. 5).

- In both retrieval methods, during the radicular access, creation of platforms, and the exposure of separated instrument’s head, the US activations in dry condition were intermittent, not exceeding 15 s [3]. This aimed to prevent heat accumulation, which could potentially cause dentinal cracks, fracture of the US tips, or secondary fractures of the separated instrument. Subsequently, the canal was irrigated with 17% EDTA to chelate dentinal debris, thereby enhancing visibility and facilitating the retrieval of the fragment [22, 23]. Additionally, this irrigation helped to cool the canal walls and reduce the temperature of the instrument fragment to avoid secondary separation [22]. The canals were then dried using paper points to prepare for the subsequent US strokes.

### Loop technique intervention

**Fig. 6:**
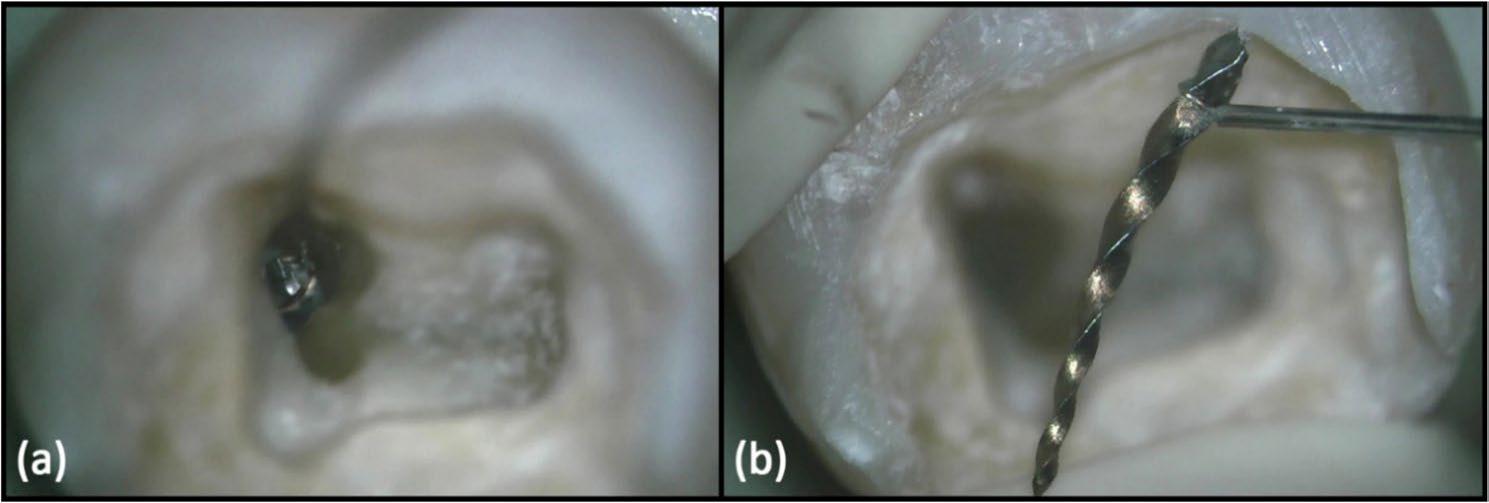
(**a**) Clinical photograph under 11x magnification of DOM showing placement and engagement of loop device (**b**) successful retrieval of 7 mm instrument fragment with BTR-Pen loop device

Samples in which retrieval was unsuccessful after 45 min; without evidence of perforation or secondary fractures, underwent a secondary intervention, using the loop technique with the BTR-Pen loop device (Cerkamed Medical Company, Stalowa Wola, Poland). The device was assembled according to manufacturer’s instructions, utilizing 0.5 mm tube diameter and 0.1 mm thick diamond wire. The wire loop was initially adjusted to the desired diameter using a DG-16 endodontic probe. The tip of the DG-16 was inserted into the loop, and loop was then tightened around it to standardize its size. The loop was then bent to approximately 45° angle relative to the long axis of the delivery tube using a cotton plier to facilitate its placement over the fractured instrument. Next, the loop was inserted into the canal with the tube positioned along the inner curvature of the canal. The wire loop was carefully guided over the exposed coronal end of the fractured instrument and oriented at approximately 90° angle over the instrument fragment, to allow complete encirclement. Visual confirmation under high magnification was mandatory to ensure full engagement of the fragment head and to prevent slippage. Once proper position was confirmed, the loop was gently tightened around the loosened fragment. A steady traction force was then applied along the long axis of the canal to withdraw the fragment. If resistance was encountered, minimal coronal manoeuvring by gentle wiggling motions was performed to disengage the fragment without applying excessive force that could risk wire fracture (Fig. 6).

Success was defined as complete fragment removal using the loop technique within a 15-minute timeframe.

### Retrieval time and success

A successful retrieval was defined as retrieval of the instrument fragment from the root canal before the assigned time elapsed. The maximum retrieval time was set to be 45 min, a reasonable chair-side duration. Time was recorded using a stopwatch, starting from the beginning of the coronal flare until the entire fractured NiTi instrument was removed, without causing secondary fractures or root perforations. Following retrieval, a #10 K-file was inserted to check for canal patency, and a final periapical radiograph was made to confirm no metal fragments were retained.

### Sample preparation for vertical root fracture resistance test

After completion of retrieval procedures, an independent researcher assigned randomized numeric codes to all specimens; the operator performing vertical root fracture testing received only these codes and remained blinded to group allocation until data collection was complete.

Each molar was sectioned vertically in a buccolingual direction through the furcation to obtain mesial and distal halves. The mesial portion was then decoronated at the cemento–enamel junction, standardizing the root length to 15 mm. All specimens were subsequently maintained at 37.5 °C and 100% humidity until vertical fracture testing, regardless of whether the separated instrument retrieval procedure had been successful or not.

The apical 5 mm of each root was covered with a uniform 0.2 mm layer of aluminium foil and then vertically embedded into a chemically cured acrylic resin block using cylindrical plastic molds (20 × 20 mm). To allow the resin to be cured fully, the blocks were left undisturbed for 24 h. After the resin blocks had completely set, the aluminium foil was removed, and the space it had occupied was filled with a light-body polyvinylsiloxane layer (Zhermack SpA, Badia Polesine, Italy) to replicate the periodontal ligament. The root was repositioned in the resin block and mechanically tested for fracture resistance employing a universal testing machine (Instron Model 3345, England). Each resin block was mounted on the device, and a 1.5-mm spherical tip was aligned vertically to contact the root center. Up until fracture, which is indicated by an abrupt decline in the force curve, load was applied at a crosshead speed of 1 mm/min. Maximum fracture loads were automatically recorded in Newtons (N) using BlueHill Universal software (Instron, England).

No samples were excluded from the analysis, as neither secondary fracture of separated instruments nor root perforations were observed.

### Statistical analysis

Statistical analyses were performed on coded, de-identified data by a statistician who was blinded to group allocation. The Shapiro-Wilk test, was used to assess the data distribution’s normality. The mean, standard deviation (SD), median, minimum, and maximum values were used to express continuous variables. For comparisons between independent samples, Mann-Whitney U test for continuous data with the Hodges-Lehmann estimator of the median difference and its 95% CI was applied. Whereas Kruskal-Wallis test followed by Dunn’s post hoc analysis was employed for comparisons involving more than two related samples. A significance threshold of *p* < 0.05 was adopted for all tests. Statistical analyses were conducted using SPSS software (IBM Corp., Released 2017, IBM SPSS Statistics for Windows, Version 25.0, Armonk, NY, USA).

## Results

Ultrasonic retrieval time results (in minutes) are presented in Table 1.

**Table 1:** Descriptive statistics and the result of Mann-Whitney U test for comparison of US retrieval time in minutes of the 3 and 5 mm fragments between the two groups and for the loop retrieval time

Technique	Fragment length		MSP group	CSP group	Median difference (HL, 95%CI)	*p*-value
*Ultrasonic retrieval*	*3 mm*	*Mean (SD)*	7.8 (1.6)	12.3 (2.3)	-4.3 (-6.0,-3.0)	<0.001*
		*Median (Range)*	7 (7–12)	12 (8–16)		0.002*
	*5 mm*	*Mean (SD)*	24 (7.5)	13 (4.9)	10.3 (5.3,18.0)	
		*Median (Range)*	24 (14–35)	14 (7–25)		
	*7 mm*	*Mean (SD)*	NA	NA		NA
		*Median (Range)*	NA	NA		
*Loop Retrieval*	*7 mm*	*Mean (SD)*	NA	1.6 (0.5)		NA
		*Median (Range)*	NA	1 (1–2)		

*Significant at *p* 0.05

Regarding the 3- and 5-mm fragments, there was a significant difference between the two groups, where MSP showed significantly lower mean values for the 3 mm fragment than the CSP *(p* < 0.001). While for the 5 mm, the CSP showed significantly lower mean value than the MSP (*p* = 0.002).

The 7 mm fragments in both groups, however, consistently required more than 45 min, exceeding the predefined time limit for analysis, and hence, no descriptive data were available.

As for the loop retrieval time, the 7 mm fragments, mean and standard deviation values were (1.6 ± 0.5) in the CSP group. For the MSP, no descriptive data were available, as all the samples failed to be retrieved by the loop within the predetermined time.

The Incidence of successful US retrieval for all 3- and 5-mm fragment samples within the MSP and CSP groups showed successful US retrieval in less than 45 min (100%). Meanwhile, all 7 mm fragment samples in both groups (100%) showed failed retrieval attempts by ultrasonic in less than 45 min.

**Fig. 7:**
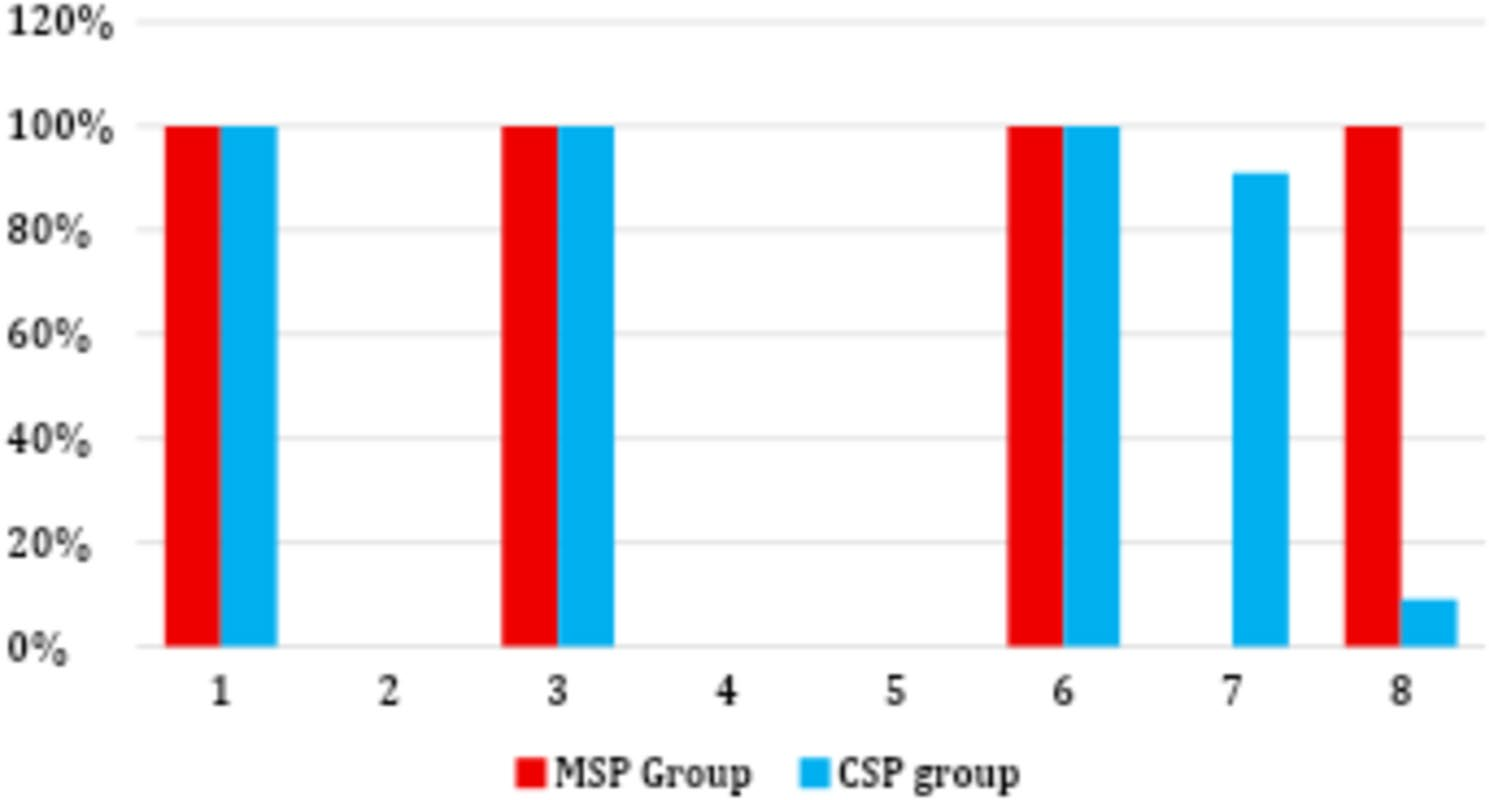
Bar chart representing the incidence of successful US retrieval of the separated fragments in the two groups

For failed 7 mm fragments, all samples (100%) in the MSP group showed failed loop retrieval, whereas in the CSP group, 10 fragments (90.9%) were successfully retrieved, while one fragment (9.1%) showed failed loop retrieval. Significant differences were evident between the two groups (*p* < 0.001) (Fig. 7).

The fracture resistance for all tested fragment lengths (3 mm, 5 mm, and 7 mm) revealed no statistically significant difference between the CSP and MSP groups; however, both groups exhibited significant differences with respect to the control group, which demonstrated the highest mean and standard deviation values for fracture resistance (*p* < 0.001).

No statistically significant differences were detected among the 3-mm, 5-mm, and 7-mm fragments in the MSP group (*p* = 0.178), noting that the 7 mm fragments were retained and not retrieved. In contrast, within the CSP group, the 3 mm fragments significantly differed from the 7 mm fragments (*p* = 0.002), while no significant differences were found between the 3 mm and 5 mm fragments or between the 5 mm and 7 mm fragments (Table 2).

**Table 2:** Descriptive statistics and the result of Kruskal-Wallis test followed by Dunn’s post hoc test for pairwise comparisons of fracture resistance (Newton -N) between the three groups

	Descriptive statistics	MSP group	CSP group	Control group	*p*-value
*3 mm*	*Mean (SD)*	301.3^b^ (95.2)	285.8^Ab^ (94)	470.4^a^ (61.5)	< 0.001*
	*Median (Range)*	302 (151–442)	244 (197–413)	462 (383–582)	
*5 mm*	*Mean (SD)*	230.3^b^ (93.6)	213.2^ABb^ (39.8)	470.4^a^ (61.5)	< 0.001*
	*Median (Range)*	190 (143–407)	216 (161–268)	462 (383–582)	
*7 mm*	*Mean (SD)*	267.5^b^ (75.2)	176.4^Bb^ (33.6)	470.4^a^ (61.5)	< 0.001*
	*Median (Range)*	242 (200–407)	169 (138–222)	462 (383–582)	
*p*-value		0.178	0.002*		

* Significant at *p* = 0.05, ** Different lower-case letters indicate statistical significance within the same row, *** Different upper-case letters indicate statistical significance within the same column

## Discussion

This study aimed to compare MSP and CSP techniques for US retrieval of 3-, 5-, and 7-mm separated instruments from moderately curved mesial roots of mandibular molars, assessing retrieval time, success rate, and vertical root fracture resistance. The results of this study contributed to a partial rejection of the null hypothesis.

Mandibular molars, namely their mesiobuccal canals, were deliberately selected in this study due to their anatomical complexity and high incidence of instrument separation reported at 55.5% of all cases, compared to 33.3% in maxillary molars [24]. Also, Anand et al., 2023 demonstrated that 48.1% of separated instruments were associated with the mesial roots of mandibular molars, reflecting the influence of their sharp curvature and narrow canals [25]. Instrument separation is further influenced by factors including instrument design, taper, and flexibility, where greater taper (e.g., 0.06) more susceptible to fracture under torsional and cyclic fatigue, especially in curved canals [24, 26]. Accordingly, the MG3 Gold file (25, 0.06) was employed as the instrument for retrieval.

Selection of fragment lengths (3, 5, and 7 mm) was based on their clinical relevance. Specifically, previous evidence establishes that fragments shorter than 4 mm are generally more amenable to ultrasonic retrieval, whereas fragments exceeding 5 mm demonstrate a significant reduction in retrieval predictability due to increased binding forces and apical engagement [27]. Therefore, evaluating these three lengths allowed assessment across predictable, borderline, and highly challenging scenarios.

Unlike previous studies that decoronated samples, to simplify access, natural crowns were preserved, maintaining anatomical constraints and improving clinical applicability [9, 16]. Moreover, a specific timeframe was allocated for each technique, US technique (45 min) and the loop technique (15 min), as advocated by Suter et al., 2005, who validated that US retrieval beyond 45–60 min shows reduced success rates and increased complications, aligning with clinical best practices [3].

The CSP technique provides access and visibility to separated instruments through straight-line drilling, which lacks selective troughing, potentially causing destructive dentin removal in curved canals, leading to excessive dentin loss and increased risks of canal transportation or perforation [10, 11, 28]. In contrast, the MSP technique has been recently introduced, employing selective troughing along the inner curve, approaching the separated instrument at a slight angle, minimizing unnecessary dentin removal while enhancing visualization and accessibility [12, 20].

The results demonstrated significantly shorter retrieval time for the 3 mm fragments with MSP when compared to CSP. Although no previous studies have compared CSP and MSP in the retrieval of 3 mm fragments in the middle third, several studies are consistent with the average retrieval time (7–16 min) observed. These results align with Madarati et al., 2010 [29] and Dulundu and Helvacioglu-Yigit, 2022 [14], who found mean time of 14.8 and 18.8 min, respectively, for separated instruments in middle third. Similarly, Kumar et al., 2021 [30] reported a broader range (11–42 min). Shahabinejad et al., 2013 [9]; reported a longer average time of 32.62 min for retrieval. Conversely, Terauchi et al., 2021 [27] reported an average of 3.7 min for retrieval. Such variability in the results might be due to root canal anatomical complexity, tooth type, and location of separated instrument [30]. From a biomechanical perspective, the significantly shorter retrieval time of 3 mm fragments with MSP might be further explained by considering variable fragment engagement. Short fragments are usually less engaged and can be removed more easily, often dislodged by irrigant turbulence and dentin debris released around their coronal threads [31]. The MSP technique, through its focused US trephination along the inner curvature, may further promote deeper irrigant penetration and enhanced acoustic streaming, facilitating quicker fragment retrieval. Collectively, this efficiency supports the growing emphasis on minimally invasive retrieval strategies and suggests that the additional dentin sacrifice required for a full CSP is not only unnecessary for short fragments but may prolong clinical time [12].

Conversely, for 5 mm fragments, the CSP demonstrated superior retrieval speed. This reversal can be explained biomechanically. As fragment length increases, its surface contact area with the canal wall also increases, particularly in the coronal and middle thirds [6]. Consequently, successful retrieval requires a more comprehensive, multi-planar loosening process. The 360° access afforded by CSP enables the ultrasonic tip to engage the fragment circumferentially, systematically disengaging it from the surrounding dentin [5, 21]. In comparison, the restricted access of the MSP becomes a limitation in this scenario, as it hinders effective engagement with the fragment’s outer wall, leading to longer operative times [12, 20]. Therefore, this result reinforces the established protocol of broad radicular access for challenging retrievals and is consistent with literature identifying fragment length as a primary determinant of difficulty [9, 32].

A 100% retrieval success rate was achieved for both 3 mm and 5 mm fragments within the clinically recommended 45-minute threshold, consistent with the findings of Cujé et al., 2010 [33] and Shahabinejad et al., 2013 [9], who achieved 100% success in middle third separated instruments retrieval. However, at 7 mm fragments, both techniques failed in the retrieval. Despite that, all 7 mm fragments in both techniques were successfully loosened following US troughing of their coronal third. Never less, none could be retrieved because the apical portion remained constrained by the canal curvature, causing the coronal end to deflect against the outer wall. These findings align with previous study indicating that fragments exceeding 5 mm in length are more challenging to retrieve using ultrasonic alone and often benefit from broader access or additional tools to enhance retrieval efficiency [27]. This finding underscores the mechanical limitation of US retrieval in curved canals for longer fragments and supports the need for adjunctive methods, such as loop devices to reduce treatment time and lower the risk of secondary fracture [29, 34].

Loop-assisted retrieval was employed for the 7 mm fragments. CSP yielded a significantly higher success rate (90.9%) compared to MSP (0%), underscoring the importance of sufficient coronal access for mechanical engagement. CSP’s 360° access facilitated loop placement, while MSP’s limited 180° troughing restricted fragment manipulation, even after ultrasonic loosening. These observations are consistent with Terauchi et al., 2021 [27] and Di Fiore et al., 2007 [35], who also highlighted the importance of sufficient coronal exposure for successful loop engagement.The failure of the MSP could be further explained by the fact that, the 180° trough frees the fragment along the inner wall, but the fragment often remains bound against the outer canal wall, preventing its coronal end from lifting into the loop’s path. This mechanical constraint underscores the limitation of US retrieval in curved canals for longer fragments and explains the inferior loop performance following MSP preparation under our standardized protocol. Importantly, this limitation can be addressed in clinical practice by using adjunctive techniques, such as placing a temporary cushion (e.g. Teflon tape) to laterally displace the fragment, thereby creating the space needed for loop engagement. Accordingly, this study’s-controlled design highlights the necessity of such advanced manoeuvres when applying the MSP to long fragments and clarifies that the CSP provides a more straightforward pathway to loop retrieval without requiring additional steps.

Since dentin removal is inevitable during instrument retrieval, all experimental groups in this study exhibited reduced fracture resistance compared to control. In agreement with previous studies, this data confirmed a significant reduction in root strength for both MSP and CSP groups, demonstrating the detrimental impact of extensive dentin removal on fracture resistance [14–16]. Although not statistically significant, the MSP group consistently showed higher mean fracture resistance values, suggesting a potential biomechanical advantage attributed to its more conservative dentin removal approach. Additionally, regaining canal patency after file retrieval was easier in the MSP group, whereas the CSP group often required precurved manual files, similar to ledge bypassing techniques, to navigate the canal and re-establish patency. The absence of significantly superior fracture resistance values in the MSP group compared to CSP may be attributed to the fact that, although MSP conserves dentin laterally through its 180° selective troughing, it often requires deeper apical extension beyond the coronal one-third of the fragment to achieve successful retrieval. This additional depth of preparation may have offset the benefits of lateral dentin preservation suggesting that the depth of troughing may be as critical as the circumference in determining residual root strength.

The MSP group with retained 7 mm instrument fragments, which could not be retrieved, demonstrated higher fracture resistance than the MSP group with retrieved 5 mm fragments. This observation may suggest that the presence of the retained fragment provided internal reinforcement, potentially contributing to greater resistance against vertical root fracture. Obturation was intentionally omitted to eliminate its potential influence on tooth fracture resistance, as previous studies have shown that both cold lateral and warm vertical compaction techniques induce dentinal defects, potentially compromising resistance to vertical root fracture [36, 37].

Taken together, these findings support a fragment-length–based clinical protocol: MSP is recommended for short fragments (3 mm), due to its time efficiency and conservative coronal exposure of separated instruments; CSP is preferable for medium fragments (5 mm), for faster retrieval via broader ultrasonic engagement, while the MSP, though slower, offers the advantages of greater dentin preservation and relatively higher fracture resistance with easier re-establishment of canal patency. For long fragments (≥ 7 mm), clinicians should anticipate ultrasonic failure and plan for early loop-assisted retrieval via a CSP. This fragment-length-based strategy enables clinicians to select the most efficient technique while acknowledging that any retrieval procedure significantly reduces root fracture resistance. Therefore, the decision to retrieve must balance the benefit of regained canal patency against the structural cost, prioritizing minimally invasive yet effective access tailored to the clinical scenario.

The findings of this in-vitro study should be interpreted considering its inherent methodological limitations. The in-vitro model, essential for comparative control, does not encompass the restricted three-dimensional operative access encountered clinically. Methodological standardization was prioritized: fracture induction via notched instruments created a reproducible experimental condition distinct from the multifactorial nature of clinical instrument failure, a single-operator protocol eliminated inter-operator variability, and the evaluation of fracture resistance employed a simplified mechanical test suitable for inter-group comparison but not for modelling the complex, long-term functional loading of a restored tooth. These constraints define the scope of the findings and highlight specific directions such as multi-operator clinical trials and fatigue-based mechanical testing for future translational research.

## Conclusions

This in vitro study supports a fragment length-based strategy for the retrieval of separated endodontic instruments. The MSP is recommended for short (3 mm) fragments, due to faster retrieval and greater dentin preservation. For moderate (5 mm) fragments, CSP provides faster retrieval, whereas the MSP, although slower, offers improved dentin preservation and canal patency. For long (7 mm) fragments, ultrasonic retrieval alone is ineffective, and successful loop-assisted removal requires complete circumferential access to instrument head, achievable with CSP. As all retrieval methods significantly reduce root fracture resistance, clinicians must carefully balance retrieval benefits against structural risks on a case-by-case basis.

## Data Availability

All data generated or analyzed during this study are included in form of tables and figures. Further information or inquiries related to the raw data are available from the corresponding author upon reasonable request ( [dina.amorsy@dentistry.cu.edu.eg](mailto: dina.amorsy@dentistry.cu.edu.eg) ) .

## Abbreviations

MSP: Modified Staging Platform
CSP: Conventional Staging Platform
NiTi: Nickel Titanium
DOM: Dental Operating Microscope
US: Ultrasonic
GG: Gates Glidden
EDTA: Ethylene-diamine-tetra-acetic acid
N: Newton
SD: Standard deviation
